# Distinct Phosphorylation Patterns of AT1R by Biased Ligands and GRK Subtypes

**DOI:** 10.3390/ijms26167988

**Published:** 2025-08-19

**Authors:** Zisu Zhang, Chuyi Liu, Jinda Gong, Chenxi Su, Zixuan Liu, Jingyuan Li, Haitao Zhang

**Affiliations:** 1 The Second Affiliated Hospital of Zhejiang University School of Medicine, Research Center for Clinical Pharmacy, Key Laboratory of Neuropharmacology and Translational Medicine of Zhejiang Province, State Key Laboratory of Advanced Drug Delivery and Release Systems, Institute of Pharmacology and Toxicology, College of Pharmaceutical Sciences, Zhejiang University, Hangzhou 310058, China; 2 School of Physics, Zhejiang University, Hangzhou 310058, China

**Keywords:** AT1R, phosphorylation, biased ligand, GRK, β-arrestin

## Abstract

G protein-coupled receptors (GPCRs) transmit through G proteins upon agonist activation, followed by phosphorylation by GPCR kinases (GRKs) to initiate β-arrestin signaling. However, the molecular mechanisms underlying GPCR signaling regulation by distinct agonists, GRK subtypes, and phosphorylation patterns remain poorly understood. The angiotensin II (AngII) type 1 receptor (AT1R), a prototypical GPCR, serves as an ideal model for studying biased ligands and signaling. Here, we investigated the wild-type (WT) AT1R and mutants of three potential phosphorylation motifs at its C-terminus (Motif I: S326/S328/S331, Motif II: T332/S335/T336/S338, and Motif III: S346/S347/S348/T349) using unbiased agonist AngII, β-arrestin-biased agonist TRV026, and G protein-biased agonist TRV056, along with GRK2/3/5/6 subtypes. We employed phosphorylation assays, β-arrestin pull-down experiments, molecular dynamics simulations, and AlphaFold3 predictions to dissect these mechanisms. Our results reveal that GRK2-mediated AT1R phosphorylation is abolished by mutations in Motifs I and II, with Motif II exhibiting a more pronounced effect. This phosphorylation was enhanced by Gβγ subunits. In contrast, GRK3-mediated phosphorylation remained unaffected by any mutations. GRK5 specifically phosphorylated Motif II, while GRK6 phosphorylated Motif II with the unbiased agonist AngII and both Motifs I and II with biased agonists TRV026 and TRV056. Notably, Motif II mutations reduced β-arrestin1/2 recruitment by GRK5/6 but not GRK2/3. Molecular dynamics simulations demonstrated that Motif II phosphorylation minimized steric hindrance, facilitating stable β-arrestin interactions, whereas Motif I phosphorylation increased intramolecular contacts that potentially impede recruitment. AlphaFold3 models provided detailed insights into the interactions between Motif II and β-arrestin1/2. Collectively, our findings elucidate diverse AT1R phosphorylation patterns driven by different agonists and GRK subtypes, offering a framework for developing signaling-biased AT1R therapeutics by decoding GRK-specific phosphorylation barcodes.

## 1. Introduction

G protein-coupled receptors (GPCRs) represent the largest family of membrane proteins in eukaryotes, with over 800 members identified in humans [1,2]. These receptors sense a wide range of extracellular stimuli, including hormones, neurotransmitters, and photons, and transduce these signals through conformational changes in their transmembrane helices to activate downstream effectors [3,4,5]. GPCR signaling regulates critical physiological processes, from synaptic transmission to cell proliferation, and their dysregulation underlies numerous diseases, including cardiovascular disorders, cancer, and metabolic syndromes [6]. Consequently, GPCRs are targeted by approximately 34% of FDA-approved drugs, highlighting their therapeutic importance [7]. GPCR signaling bifurcates into two primary pathways: G protein-dependent and arrestin-dependent signaling [5]. Following G protein activation, GPCR kinases (GRKs) phosphorylate serine/threonine residues in the receptor’s C-terminal tail (CT), promoting β-arrestin binding while attenuating G protein coupling, a process known as receptor desensitization [3,8,9]. The phosphorylation pattern on the receptor’s tail influences β-arrestin binding and can induce distinct receptor conformations, thereby shaping downstream signaling outcomes [10,11,12,13].

The angiotensin II (AngII) type 1 receptor (AT1R), a Class A GPCR, is a key component of the renin–angiotensin system (RAS) [14]. Expressed predominantly in cardiovascular cells, AT1R mediates the physiological and pathological effects of AngII, including vasoconstriction, blood pressure regulation, and renal sodium reabsorption [15,16,17,18,19]. AT1R overactivation drives pathological processes such as vascular sclerosis, organ fibrosis, and chronic kidney disease, making it a critical therapeutic target [19,20,21,22,23]. The Gq-dependent pathway of AT1R mediates physiological effects like blood pressure elevation, and the β-arrestin-dependent pathway enhances myocardial contractility and promotes cardiomyocyte hyperplasia [24,25,26,27]. This functional divergence has spurred interest in β-arrestin-biased agonists, which offer therapeutic potential for conditions such as congestive heart failure with reduced side effects [28].

Recent studies have revealed that AngII, the endogenous agonist of AT1R, activates both G protein and β-arrestin pathways without bias, whereas synthetic analogs of AngII exhibit pathway-specific agonism [29]. For instance, TRV055 and TRV056 are Gq-biased agonists, while TRV026 and TRV027 are β-arrestin-biased agonists [30,31]. Notably, AngII requires cooperative phosphorylation of AT1R by multiple GRKs (GRK2, GRK3, GRK5, and GRK6) to recruit β-arrestin, whereas the β-arrestin-biased agonist TRV027 achieves β-arrestin recruitment primarily through GRK5/6-mediated phosphorylation [32]. This suggests that biased signaling may not only depend on ligand-induced receptor conformational changes but also on GRK subtype-specific phosphorylation patterns, including site specificity and stoichiometry, which could dictate downstream signaling bias. The differential roles of GRKs in β-arrestin recruitment and activation have been increasingly elucidated. GRK5/6, but not GRK2/3, mediates β-arrestin pre-coupling to AT1R independent of AngII activation, a phenomenon attributed to receptor membrane localization and conformational specificity [33]. Furthermore, β-arrestin2 exhibits a greater capacity to probe both inactive and active receptor conformations compared to β-arrestin1, even in the absence of phosphorylation or receptor activation. This functional divergence arises from the intrinsically disordered C-terminal domain of β-arrestin2, which may facilitate autoactivation without requiring engagement of phosphorylated intracellular receptor motifs [34]. These findings underscore the intricate interplay between ligand-specific receptor conformations, GRK-mediated phosphorylation, and β-arrestin functionality, providing critical insights into the molecular mechanisms underlying biased signaling at AT1R. Understanding these mechanisms is essential for the development of targeted therapeutics that exploit pathway-specific signaling to achieve desired physiological outcomes with minimized off-target effects.

Recent advances in understanding how GPCR phosphorylation patterns regulate β-arrestin conformation and signaling selectivity have been framed by three mechanistic hypotheses: the “barcode hypothesis”, the “flute model”, and the “phosphorylation code hypothesis” [12,33,35,36,37]. The phosphorylation code hypothesis, which emphasizes the role of specific motifs PxPxxP/PxxPxxP (where “P” denotes S/T and “x” represents any amino acid) in dictating β-arrestin binding and signaling, provides a framework for dissecting the molecular mechanisms underlying GPCR signaling bias. Based on the phosphorylation code hypothesis (PxPxxP/PxxPxxP motifs), we classified the serine/threonine (S/T) residues within the AT1R C-terminal tail into three motifs for phosphorylation (Motif I: 326-SHSNLS-331, Motif II: 332-TKMSTLS-338, and Motif III: 346-SSST-349). Using AngII (unbiased), TRV026 (β-arrestin-biased), and TRV056 (Gq-biased) agonists, we investigated GRK2/3/5/6-mediated phosphorylation patterns of AT1R and their impact on β-arrestin binding.

Our findings reveal that specific phosphorylation motifs within the C-terminal tail of AT1R act as molecular switches, dictating the recruitment and activation of β-arrestin isoforms and thereby shaping downstream signaling outcomes. Additionally, we utilized molecular dynamics (MD) simulations and AlphaFold3 (AF3) to model the conformational changes induced by phosphorylation and their impact on β-arrestin binding. Our study advances the understanding of how phosphorylation patterns regulate GPCR signaling and highlights the potential for targeting specific phosphorylation motifs to achieve pathway-selective modulation of receptor activity. These findings have broad implications for the development of biased agonists and other therapeutic strategies aimed at fine-tuning GPCR signaling for the treatment of cardiovascular and other diseases.

## 2. Results

### 2.1. GRKs Exhibit Subtype-Specific Phosphorylation Preferences Induced by AngII

**Figure 1:**
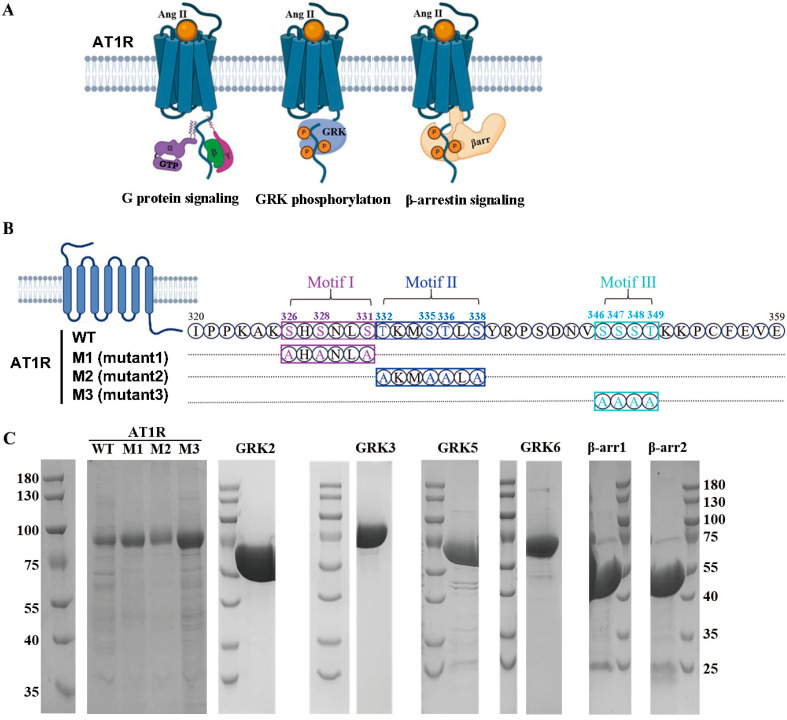
Schematic overview of AT1R signaling pathways, construct design, and protein purification for phosphorylation assays. (**A**) Schematic diagram illustrating AT1R signaling pathways through G proteins, GRK, and β-arrestin. (**B**) Delineation of AT1R C-terminal potential phosphorylation motifs. Three predicted phosphorylation motifs are colored in purple for Motif I, blue for Motif II, and cyan for Motif III, according to the phosphorylation code hypothesis. Serine/threonine (S/T) residues within the three motifs were mutated to alanine (**A**), designated as mutants M1, M2, and M3, respectively. (**C**) SDS-PAGE results of AT1R WT/mutants, four GRK subtypes, and two β-arrestin isoforms for protein purification.

Within the β-arrestin signaling pathway of AT1R, GRK-mediated phosphorylation of the AT1R C-terminal tail is critically required [38] (Figure 1A). The intrinsically disordered C-terminal region of AT1R (residues 320–359) contains multiple potential serine/threonine (S/T) phosphorylation sites. Guided by the PxPxxP/PxxPxxP phosphorylation code hypothesis, we classified these residues into three potential phosphorylation motifs: Motif I (326-SHSNLS-331), Motif II (332-TKMSTLS-338), and Motif III (346-SSST-349). To dissect the contribution of each motif, we mutated all S/T residues within each motif to alanine (A), generating three mutants, M1 (S326A/S328A/S331A), M2 (T332A/S335A/T336A/S338A), and M3 (S346A/S347A/S348A/T349A) (Figure 1B). The proteins of wild-type (WT) and mutated AT1R, as well as four GRK subtypes (GRK2/3/5/6), were individually purified (Figure 1C), reconstituted into a phosphorylation system with the endogenous balanced agonist AngII, and *in vitro* phosphorylation was detected.

**Figure 2:**
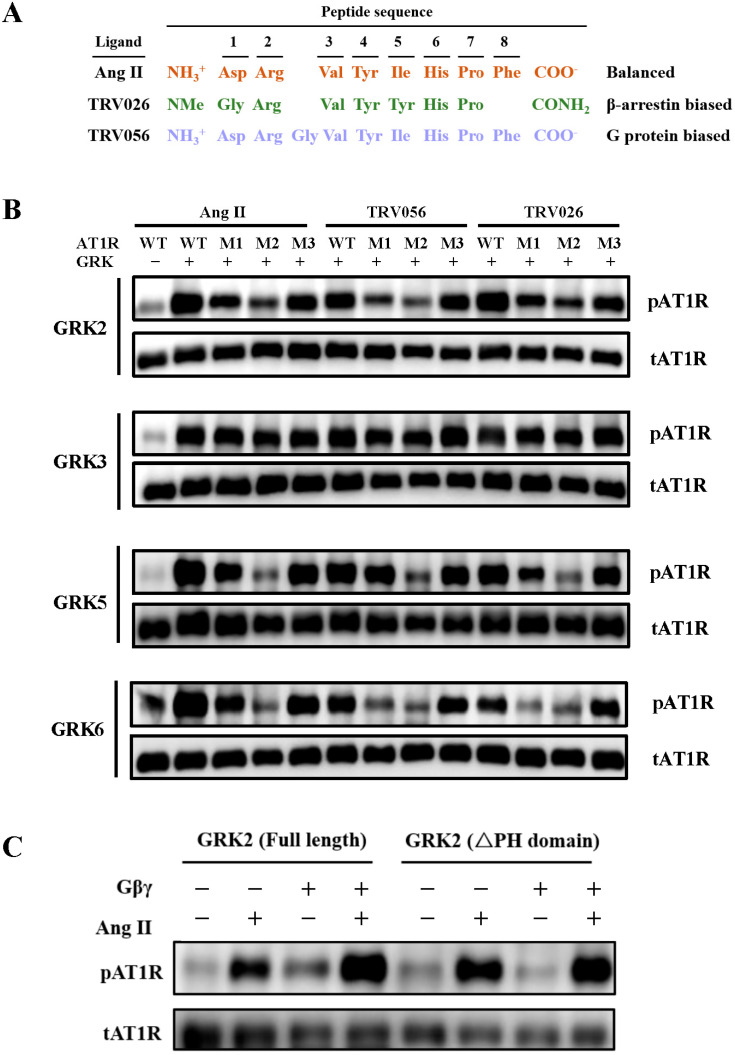
AT1R displays diverse phosphorylation patterns induced by different agonists and GRK subtypes. (**A**) Peptide sequences and signaling profiles of AngII, TRV026, and TRV056. (**B**) *In vitro* phosphorylation results of AT1R WT and M1/2/3 mutants by three agonists and four GRK subtypes. (**C**) GRK2-mediated AT1R phosphorylation facilitated by the Gβγ subunit through its PH domain.

Our phosphorylation results revealed that all four GRKs effectively phosphorylated AT1R-WT compared to the GRK-free negative control (Figure 2, Supplementary Figure S1). GRK2 exhibited the lowest phosphorylation levels for M2, reduced phosphorylation for M1, and no significant reduction for M3, indicating its primary reliance on Motif II. GRK3 showed no pronounced differences across WT and mutants, suggesting that it may phosphorylate alternative sites outside the C-terminal region. Indeed, previous studies have highlighted the importance of intracellular loops (ICLs) of AT1R in GRK/β-arrestin signaling [37]. GRK5/6 displayed markedly reduced phosphorylation for M2, with moderate decreases for M1 and M3, further underscoring Motif II as their dominant phosphorylation site.

Our *in vitro* phosphorylation approach effectively dissected GRK subtype-specific phosphorylation of AT1R, circumventing potential interference from other intracellular signaling pathways and cell line-specific post-translational modifications. Collectively, Motif II (332-TKMSTLS-338) emerged as the critical phosphorylation site for GRK2/5/6 under endogenous AngII activation, as its mutation significantly attenuated AT1R phosphorylation. These findings highlight the nuanced roles of GRK subtypes in encoding ligand-specific signaling bias through selective phosphorylation patterns, providing mechanistic insights into the regulation of AT1R signaling by the GRK/β-arrestin pathway.

### 2.2. TRV026 and TRV056 Induce Distinct Phosphorylation Patterns Compared to AngII

To compare the phosphorylation patterns induced by the unbiased agonist AngII and biased agonists, we selected the G protein-biased agonist TRV056 and the β-arrestin-biased agonist TRV026 for our study. TRV056 incorporates an additional glycine following position R2 of AngII, while TRV026 lacks the phenylalanine at F8 of AngII (Figure 2A).

For GRK2/3, the phosphorylation patterns were consistent across all agonists (Figure 2B, Supplementary Figure S1A). GRK2 exhibited reduced phosphorylation levels for both M1 and M2, with M2 showing a more pronounced reduction, indicating that GRK2 predominantly phosphorylates AT1R at Motif II. In contrast, GRK3 showed no significant differences in phosphorylation levels between WT and the M1/M2/M3 mutants, suggesting that GRK3 phosphorylates sites outside the C-terminal motifs.

Interestingly, GRK2-mediated phosphorylation of GPCRs was found to be modulated by the βγ subunit of G proteins through the formation of a GPCR-GRK2-Gβγ ternary complex [39]. The C-terminal pleckstrin homology (PH) domain of GRK2 (residues from 559 to 661), which anchors the kinase to the membrane and interacts with Gβγ, plays a critical role in this process [40,41]. To investigate the contributions of Gβγ and the PH domain to GRK2 activity, we co-expressed AT1R, Gβγ, and GRK2 proteins and assessed phosphorylation levels. Full-length GRK2 exhibited enhanced phosphorylation of AT1R in the presence of Gβγ, whereas deletion of the PH domain (ΔPH) abolished this effect (Figure 2C, Supplementary Figure S1B). These results underscore the importance of the Gβγ subunit and the PH domain in regulating GRK2-mediated phosphorylation.

For GRK5, the phosphorylation patterns induced by TRV056 were similar to those observed with AngII, with the M2 mutant showing significantly reduced phosphorylation (Figure 2B, Supplementary Figure S1A). However, TRV026 led to a substantial reduction in phosphorylation for the M1 mutant, albeit less pronounced than for M2, suggesting that Motif II remains the primary phosphorylation site for GRK5 regardless of the agonist.

In contrast, GRK6 phosphorylation patterns diverged markedly among biased and unbiased agonists. Both TRV056 and TRV026 induced a dramatic reduction in phosphorylation for the M1 mutant, compared to AngII, indicating that GRK6 phosphorylates both Motif I and Motif II in response to biased agonists (Figure 2B, Supplementary Figure S1A). This divergence highlights the ability of biased ligands to redirect GRK6-mediated phosphorylation to distinct sites on the AT1R C-terminal tail.

Collectively, these results demonstrate that different agonists and GRK subtypes induce diverse phosphorylation patterns at the AT1R C-terminal tail. While GRK2/5 predominantly target Motif II, GRK6 exhibits a more flexible phosphorylation pattern, targeting both Motif I and Motif II in response to biased ligands. GRK3, however, appears to phosphorylate sites outside the canonical C-terminal motifs. This study also reveals the critical role of the Gβγ subunit and the PH domain in regulating GRK2-mediated phosphorylation, providing mechanistic insights into how G protein signaling influences GRK activity. Furthermore, the distinct phosphorylation patterns induced by TRV056 and TRV026 underscore the ability of biased ligands to encode ligand-specific signaling bias through selective GRK activation and site-specific phosphorylation.

### 2.3. Distinct Phosphorylation Patterns of AT1R by GRKs Regulate β-Arrestin Recruitment

To investigate the role of GRK subtypes in regulating β-arrestin 1/2 recruitment to AT1R, we employed pull-down assays to quantify AT1R-β-arrestin 1/2 binding. Purified AT1R, GRK2/3/5/6, and β-arrestin 1/2 proteins were incubated with AngII, and the interactions were analyzed.

**Figure 3:**
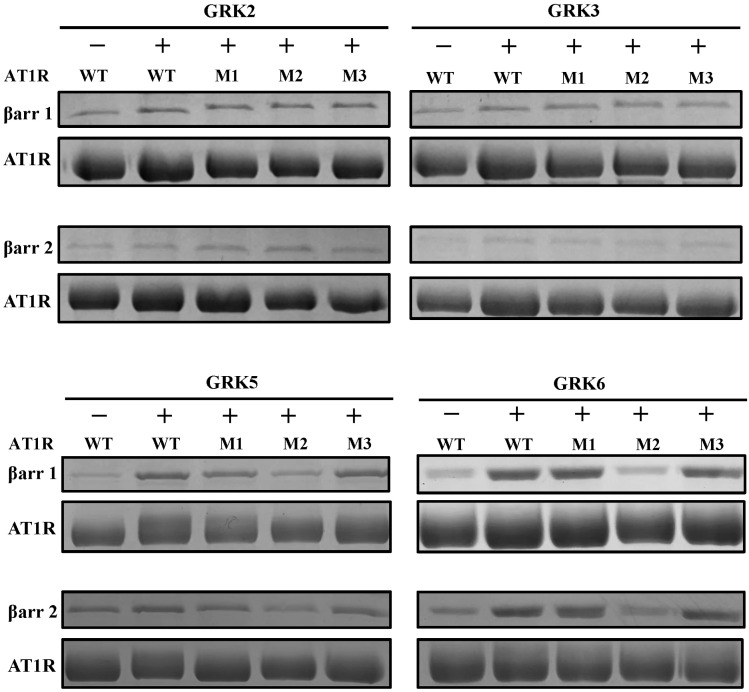
Effects of different GRKs on β-arrestin recruitment to AT1R. Pull-down assay results of β-arrestin 1/2 binding to AT1R WT and M1/2/3 mutants induced by GRK2/3/5/6 with AngII stimulation.

GRK2/3 did not significantly enhance β-arrestin 1/2 recruitment to WT AT1R (Figure 3, Supplementary Figure S2). Furthermore, mutations in the C-terminal phosphorylation motifs of AT1R (M1, M2, and M3) did not abolish β-arrestin 1/2 binding, suggesting that GRK2/3-mediated phosphorylation is not a primary driver of β-arrestin recruitment under these conditions. Previous studies have indicated that GRK2/3-dependent β-arrestin recruitment may require the involvement of G protein Gβγ subunits [32,42], highlighting a potential regulatory mechanism that was not fully recapitulated in our experimental setup.

However, GRK5/6 significantly enhanced β-arrestin 1/2 recruitment to WT AT1R (Figure 3, Supplementary Figure S2). Notably, the M2 mutant, which disrupts Motif II, exhibited dramatically diminished β-arrestin 1/2 binding, indicating that GRK5/6-mediated phosphorylation of Motif II is essential for stabilizing AngII-induced AT1R-β-arrestin complexes. These findings establish Motif II as the primary phosphorylation site for GRK5/6 and a critical structural determinant for β-arrestin recruitment.

Collectively, these results demonstrate that GRK subtypes differentially regulate β-arrestin recruitment to AT1R. While GRK2/3 have minimal effects on β-arrestin binding, GRK5/6 play a pivotal role in stabilizing AT1R-β-arrestin complexes through phosphorylation of Motif II.

### 2.4. Phosphorylation Patterns Induce Distinct Conformational Dynamics of AT1R

**Figure 4:**
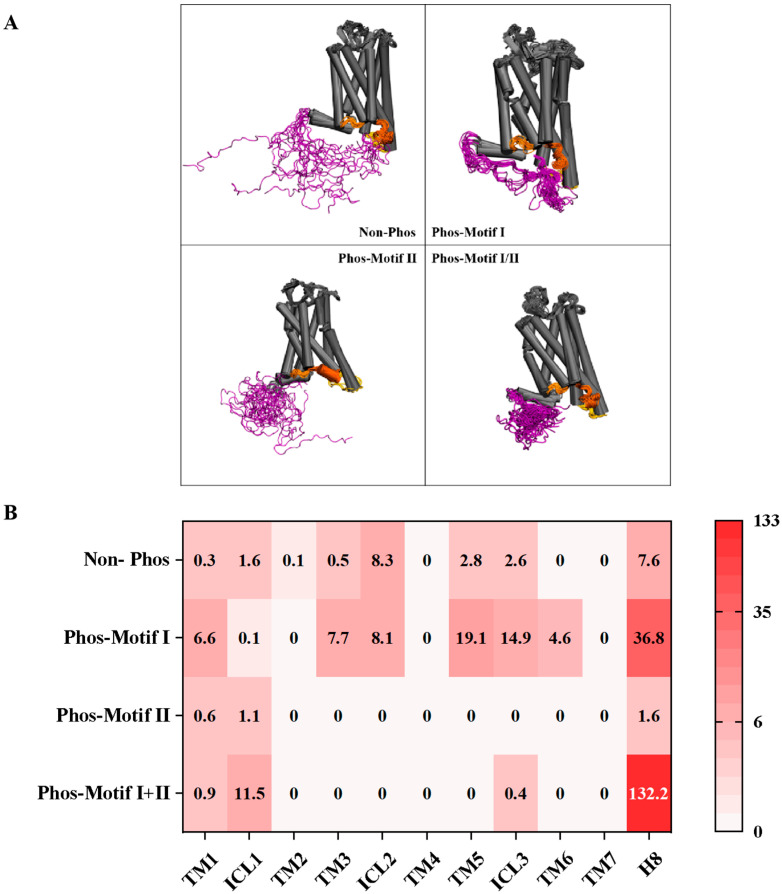
Different phosphorylation patterns exhibit different interactions between ICLs/TMs/helix8 and CT of AT1R. (**A**). Molecular dynamics simulations of the intrinsically disordered ICLs/TMs/helix8 and CT corresponding to four different phosphorylation patterns of AT1R. Purple denotes the AT1R CT fragment, orange-yellow denotes ICL1, orange-red denotes ICL2, and yellow denotes ICL3. (**B**) Heatmap of the contact matrix of AT1R CT with ICLs/TMs/helix8 in different phosphorylation modes. A darker red color indicates a larger area of AT1R-CT in contact with the region.

To explore how different phosphorylation patterns of AT1R C-terminus influence β-arrestin interactions, we constructed four molecular dynamics (MD) simulation systems based on our previously solved Sar^1^-AngII-AT1R-Gq cryo-electron microscopy (cryo-EM) structure [Protein Data Bank (PDB) code: 7F6G] [43,44]. These systems represented the non-phosphorylated state, Motif I-phosphorylated, Motif II-phosphorylated, and dual phosphorylation at both Motifs I and II. Our MD simulations revealed phosphorylation-dependent alterations in the dynamics of transmembrane helices (TM1-7), intracellular loops (ICL1-3), helix8, and the C-terminus, with corresponding contact matrices quantifying interface interactions under each condition (Figure 4).

The ICLs, helix8, and CT of GPCRs are critical for G protein and β-arrestin engagement. In the non-phosphorylated state, the ICLs/helix8/CT fragments of AT1R exhibited intrinsically disordered distribution with limited contact surfaces. Phosphorylation of Motif I induced structural ordering of the CT fragment and enhanced interactions with ICL2/3 and helix8, potentially hindering β-arrestin binding. In contrast, phosphorylation of Motif II reduced ICL-CT contact surfaces to near-background levels, preserving β-arrestin binding capacity. Dual phosphorylation at both Motifs I and II preferentially directed CT interactions toward ICL1 and helix8, further influencing β-arrestin association.

These findings elucidate the phosphorylation pattern-specific reorganization of the CT fragment’s spatial arrangements. Notably, phosphorylation of Motif I increases AT1R ICLs/helix8/CT interaction surfaces, potentially inhibiting β-arrestin binding, whereas phosphorylation of Motif II maintains minimal interference with binding interfaces. This validates Motif II as the primary determinant regulating AT1R-β-arrestin interactions. Our results thus demonstrate that distinct phosphorylation patterns of AT1R induce specific conformational dynamics, which in turn modulate β-arrestin binding.

### 2.5. AlphaFold3 Predicts Phosphorylation-Dependent Mechanisms of AT1R-β-Arrestin Complex Formation

**Figure 5:**
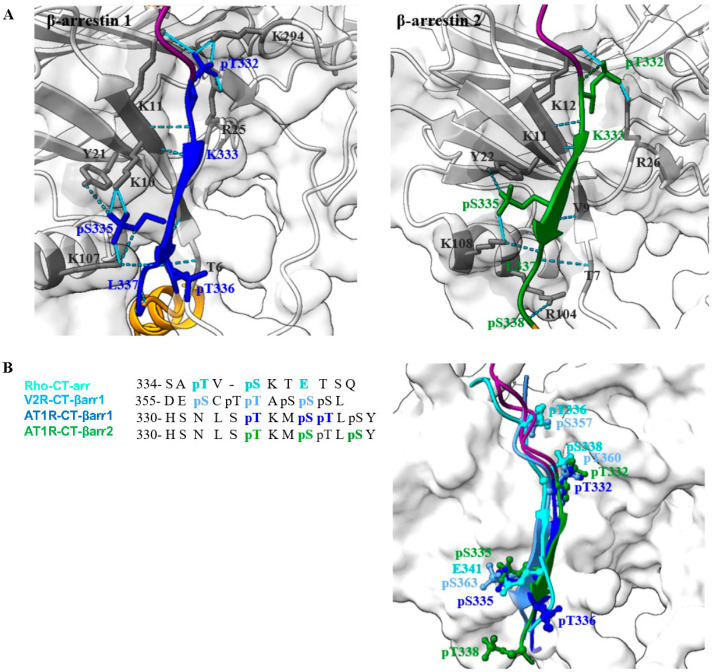
AlphaFold3 predicted models of AT1R-β-arrestin for phosphorylation mechanisms. (**A**) Predicted interactions of phosphorylated AT1R CT and β-arrestin1/2. The β-arrestin is shown in carbon color, with Motif I in purple and Motif II in blue/green. Hydrogen bonds are represented by dashed lines, while salt bridges are indicated by solid lines. (**B**) Structure-based sequence alignments of phosphorylated AT1R, V2R, and rhodopsin CTs (PDB codes: 4JQI and 5W0P).

To elucidate the structural mechanisms underlying AT1R-β-arrestin interactions, we employed AlphaFold3 (AF3) to predict models of AT1R-β-arrestin complexes in the presence of Motifs I and II phosphorylation [45,46,47,48]. The AF3 models revealed distinct roles for phosphorylated Motifs I and II in mediating β-arrestin binding (Figure 5A). Phosphorylated Motif II exhibited extensive interactions with both β-arrestin1 and 2. In the AT1R-β-arrestin1 complex, pT332 formed salt bridges with K11, R25, and K294 of β-arrestin1, while pS335 engaged in salt bridges with K10 and K107. Additionally, pT336 formed a hydrogen bond with K107. In the AT1R-β-arrestin2 complex, pT332 formed salt bridges with K12 and R26 of β-arrestin2, pS335 interacted with K108 via a salt bridge and Y22 via a hydrogen bond, and pS338 formed a hydrogen bond with R104. These interactions highlight the critical role of phosphorylated Motif II in stabilizing AT1R-β-arrestin complexes. In contrast, phosphorylated Motif I exhibited no significant interactions with β-arrestin1/2, confirming that Motif I phosphorylation is not essential for AT1R-β-arrestin binding.

Structural analysis revealed conserved PxxPP and PxxPxxP motifs within the β-arrestin1/2 interaction interfaces of the AT1R C-terminus. Structure-based sequence alignments of the AT1R CT with experimentally determined structures of the vasopressin receptor V2R and rhodopsin demonstrated the prevalence of β-sheet conformations in phosphorylated receptor CTs (Figure 5B) [11,49]. This suggests a conserved structural mechanism for GPCR-β-arrestin interactions.

The AF3-predicted models demonstrate that interactions between AT1R and β-arrestin1/2 are predominantly mediated by phosphorylated Motif II, which forms extensive salt bridges and hydrogen bonds with key residues in β-arrestin1/2. In contrast, phosphorylated Motif I plays no significant role in these interactions. These findings provide a structural basis for understanding how phosphorylation patterns regulate AT1R-β-arrestin complex formation and highlight the conserved nature of these mechanisms across GPCRs.

## 3. Discussion

Our study systematically investigates the regulatory mechanisms of different GRK subtypes (GRK2/3/5/6) on AT1R phosphorylation patterns induced by distinct agonists (AngII/TRV026/TRV056) and their impacts on β-arrestin1/2 recruitment, revealing the complexity and subtype specificity of phosphorylation coding. By applying the phosphorylation barcode theory, we divided the intrinsically disordered C-terminal region of AT1R into three critical phosphorylation clusters: Motif I (326-SHSNLS-331), Motif II (332-TKMSTLS-338), and Motif III (346-SSST-349). Through alanine mutagenesis experiments, we identified distinct phosphorylation preferences among GRK subtypes, providing mechanistic insights into their roles in AT1R signaling.

GRK2 primarily phosphorylated Motif II when AT1R was activated by the endogenous balanced agonist AngII, while Motif I played a modest role in GRK2-mediated phosphorylation. In contrast, GRK3 exhibited no pronounced site selectivity, suggesting its phosphorylation mechanism may operate independently of specific C-terminal sequences or rely on conformational changes in other receptor regions, such as intracellular loops (ICLs). GRK5/6, however, preferentially targeted Motif II, highlighting their role as core regulators of AT1R-β-arrestin signaling pathways. Notably, GRK6 exhibited remarkable ligand-dependent phosphorylation patterns, in which it preferentially phosphorylated Motif I in the presence of AngII but simultaneously targeted both Motifs I and II when stimulated by the biased agonists TRV056 and TRV026. These findings suggest that ligand bias fine-tunes receptor phosphorylation patterns, modulating signal transduction efficiency.

Phosphorylation assays with Gβγ demonstrated that Gβγ binding to the GRK2 PH domain enhances its kinase activity, a mechanism abolished upon PH domain truncation. This underscores the essential role of G protein subunits in GRK2 phosphorylation activity. Furthermore, GRK2/3-mediated phosphorylation consistently induced low-level β-arrestin binding across all phosphorylation site mutants, indicating that their phosphorylation patterns were insufficient for full β-arrestin activation or complex stabilization. Emerging evidence suggests that beyond the C-terminal disordered region, the AT1R ICLs may play critical roles in GRK2/3-β-arrestin pathway regulation. Additionally, phosphorylation patterns promoting β-arrestin recruitment may differ from those enabling its activation, implying that understanding AT1R signaling requires consideration of full-receptor conformational changes and detailed mechanistic characterization of each step in the signaling cascade.

GRK5/6-mediated phosphorylation, particularly at Motif II, significantly enhanced β-arrestin recruitment, establishing these kinases as key regulators of AT1R-β-arrestin signaling. In contrast, GRK2/3-mediated phosphorylation induced only weak β-arrestin binding, suggesting their primary role in G protein pathway desensitization rather than β-arrestin pathway activation.

To further elucidate the structural basis of phosphorylation-dependent β-arrestin recruitment, we employed molecular dynamics simulations to investigate conformational changes in the AT1R C-terminal region under different phosphorylation patterns. Phosphorylation at Motif I increased the contact area between the CT and ICLs, potentially hindering β-arrestin recruitment. In contrast, phosphorylation at Motif II reduced CT-ICLs interactions, eliminating steric hindrance and facilitating AT1R-β-arrestin binding. These findings highlight Motif II as the primary phosphorylation site for AT1R-β-arrestin interactions.

Finally, we utilized AlphaFold3 to predict protein–protein interactions and systematically elucidate the interactions of phosphorylated AT1R Motif I and Motif II with β-arrestin1/2. Our analysis revealed that phosphorylated Motif I does not interact with β-arrestin1/2, suggesting it is not a key structural unit for β-arrestin recruitment. In contrast, phosphorylated Motif II exhibited significant interaction features, with characteristic PxPxxP/PxxPxxP motifs at the binding interface. These findings align with experimentally determined structures of V2R/rhodopsin-arrestin complexes, further validating the conserved nature of these interactions.

Recent studies employing mass spectrometry (MS), bioluminescence resonance energy transfer (BRET), and enzyme complementation-based (DiscoverX) endocytosis assays in Expi293F/HEK293 cells have elucidated AT1R phosphorylation *in vivo* [46]. These investigations demonstrated that both the endogenous agonist AngII and the β-arrestin-biased agonist TRV023 enhance phosphorylation of the AT1R C-terminal tail, with AngII inducing more pronounced phosphorylation in the proximal region (designated as Motif I in our study). Furthermore, phosphorylation of both proximal and middle regions (Motifs I and II) was shown to be essential for β-arrestin recruitment and receptor internalization. However, the effects of G protein-biased agonists and GRK subtypes were not comprehensively examined. In this study, we extended these findings by investigating the Gq protein-biased agonist TRV026 and four GRK subtypes (GRK2/3/5/6), revealing their distinct regulatory roles in AT1 phosphorylation *in vitro*. Our results demonstrate that GRK2-mediated AT1R phosphorylation is abolished by mutations in Motifs I and II, with Motif II exhibiting a more substantial impact. This phosphorylation process was enhanced by Gβγ subunits. In contrast, GRK3-mediated phosphorylation remained unaffected by any mutations. GRK5 specifically phosphorylated Motif II, while GRK6 phosphorylated Motif II in the presence of the unbiased agonist AngII and both Motifs I and II with biased agonists TRV026 and TRV056.

While our study provides critical insights into GRK-mediated AT1R phosphorylation and β-arrestin recruitment, several limitations and open questions remain. First, the *in vitro* reconstitution system may not fully recapitulate the dynamic cellular environment of AT1R-GRK interactions, necessitating further cell-based experiments to validate the functional relevance of phosphorylation patterns. Second, although the C-terminal disordered region is the primary site for phosphorylation, the role of ICL phosphorylation in β-arrestin activation remains underexplored and warrants further investigation. Third, the detailed molecular interactions between AT1R and GRKs, particularly under different agonist-induced receptor conformations, require experimental structure determination to fully elucidate the mechanisms underlying GRK phosphorylation selectivity. Additionally, the functional consequences of phosphorylation patterns beyond β-arrestin recruitment, such as their impact on receptor internalization, recycling, and downstream signaling pathways, remain to be explored. Future studies should also investigate the interplay between GRK-mediated phosphorylation and other post-translational modifications, such as ubiquitination or palmitoylation, which may further modulate AT1R signaling dynamics.

Our findings underscore the complexity of GRK-mediated phosphorylation coding and its role in fine-tuning GPCR signaling. The subtype-specific phosphorylation patterns and their differential effects on β-arrestin recruitment highlight the importance of considering GRK diversity for the development of biased agonists and therapeutic strategies targeting AT1R. By elucidating the structural and mechanistic basis of AT1R phosphorylation, our study provides a framework for understanding how phosphorylation barcodes regulate receptor signaling and offers insights into the design of more precise pharmacological interventions.

In summary, our study systematically dissects the regulatory mechanisms of GRK subtypes in AT1R phosphorylation and β-arrestin recruitment, revealing the complexity and specificity of phosphorylation coding. Our findings highlight the critical roles of GRK5/6 in β-arrestin pathway activation and the distinct contributions of GRK2/3 in G protein pathway desensitization. By integrating mutagenesis, MD simulations, and AlphaFold3-based protein interaction predictions, we provide a comprehensive understanding of the structural and functional consequences of AT1R phosphorylation. These insights advance our knowledge of GPCR signaling and pave the way for future investigations into the broader implications of phosphorylation barcodes in GPCR biology and drug discovery.

## 4. Materials and Methods

### 4.1. AT1R Protein Engineering and Cloning

To facilitate the protein expression and purification, an HA signaling peptide, a FLAG tag, a 10 × His tag, and a maltose-binding protein (MBP) tag were fused to the N terminus of AT1R. Site-directed mutagenesis was performed to generate phosphorylation-deficient mutants of AT1R by targeting three regions at its C-terminal tail, designated as Motif I, Motif II, and Motif III. All serine and threonine residues within each motif were substituted with alanine and denoted as M1, M2, and M3. The mutants were constructed using overlap extension PCR with the following primer pairs with wild-type (WT) AT1R as the template. M1: Forward primer: 5′-GCTAAGGCCCACGCCAACTTGGCC-ACTAAG-3′; Reverse primer: 5′-CTTAGTGGCCAAGTTGGCGTGGGCCTTAGC-3′. M2: Forward primer: 5′-TTGTCCGCCAAGATGGCCGCCCTGGCCTACCGC-3′; Reverse primer: 5′-GCGGTAGGCCAGGGCGGCCATCTTGGCGGACAA-3′. M3: Forward primer: 5′-GATAACGTCGCCGCCGCCGCCAAGAAGCCC-3′. Reverse primer: 5′-GGG-CTTCTTGGCGGCGGCGGCGACGTTATC-3′. The full-length human AT1R cDNA was subcloned into the pFastBac1 vector (Invitrogen, Carlsbad, CA, USA) for subsequent recombinant bacmid generation and protein expression. All mutations were verified by bidirectional Sanger sequencing prior to functional analyses.

### 4.2. AT1R Protein Expression and Purification

AT1R was expressed in *Spodoptera frugiperda* (*Sf*9) insect cells (Thermo Fisher, Agawam, MA, USA). Cells were grown in ESF921 medium (Expression Systems, Davis, CA, USA) and infected with virus simultaneously at MOI = 5 when the cell density reached ~2.1 × 10^6^ cells/mL. After 48h of infection, cells were harvested. Cell pellets were thawed on ice and lysed by Dounce homogenization in low-salt lysis buffer (10 mM HEPES pH 7.5, 20 mM KCl, 2 mM MgCl_2_), supplemented with protease inhibitor cocktail (500 µM AEBSF, 1 µM E-64, 1 µM leupeptin, and 150 nM aprotinin). After centrifugation at 58,000× *g* for 30 min, cell pellets were homogenized with high salt lysis buffer (10 mM HEPES pH 7.5, 1 M NaCl, 20 mM KCl, 10 mM MgCl_2_) and then centrifuged at 58,000× *g* for 30 min. This experimental procedure was repeated twice. Membranes were resuspended in low-salt lysis buffer, containing 50 µM ligand (Genscript, Ridgefield, CT, USA) and 2 mM iodoacetamide, followed by incubation at 4 °C. Membranes were solubilized at 4 °C for 3 h in buffer supplemented with 0.5% (*w*/*v*) lauryl maltose neopentyl glycol (LMNG, Anatrace, Maumee, OH, USA) and 0.05% (*w*/*v*) cholesteryl hemisuccinate Tris salt (CHS, Anatrace, Maumee, OH, USA). The lysate was clarified by ultracentrifugation at 58,000 g for 1 h at 4 °C. The supernatant was incubated with Talon IMAC resin (Takara, Shiga, Japan) overnight at 4 °C, and the resin was subsequently loaded onto a gravity column (Bio-Rad, Hercules, CA, USA). Bound protein was washed with 20 column volumes (CVs) of wash buffer (50 mM HEPES, pH 7.5, 0.05% (*w*/*v*) LMNG, 0.005% (*w*/*v*) CHS, 20 mM imidazole, and 10 μM ligand). With 10 CV of elution buffer (20 mM HEPES, pH 7.5, 100 mM NaCl, 0.01% (*w*/*v*) LMNG, 0.001% (*w*/*v*) CHS, 300 mM imidazole, and 50 μM ligand). Purified AT1R was concentrated to the appropriate concentration for the next step.

### 4.3. GRK/β-Arrestin Protein Expression and Purification

Through systematic screening of protein expression and stability across various cellular and vector systems, GRK5/β-arrestin1/2 were expressed in *E*. *coli*, and GRK2/3/6 were expressed in *Sf*9 insect cells, with a 6×His tag for purification at the C-terminus. GRK5 has the T10A mutation, truncation at position 561 to remove its autophosphorylation sites, and the VD dipeptide at the C-terminus to achieve high expression levels while maintaining high enzymatic activity [50]. To capture the activated state of the AT1R-β-arrestin complex and better complex homogeneity while maintaining functional integrity, the β-arrestin1 was engineered with the activating mutations I386A/V387A/F388A, the 7C stabilizing mutations C59V/C125S/C140L/C150V/C242V/C251V/C269S, and the C-terminal truncation at residue 393, while β-arrestin2 was modified with the 7C stabilizing mutations C59V/C125S/C140L/C150V/C242V/C251V/C269S and C-terminal truncation at residue 393 [51,52]. The GRK5 plasmid was transformed into BL21(DE3) cells and cultured in LB medium. When the OD_600_ reached 0.4–0.6, protein expression was induced with 0.5 mM IPTG at 20 °C for 20 h. Bacterial pellets were harvested by centrifugation and resuspended in lysis buffer (20 mM HEPES, 250 mM NaCl, 0.02% Triton X-100, and 0.1 μM TCEP). The suspension was sonicated on ice for 20 min and centrifuged at 30,000× *g* for 1 h. The supernatant was incubated with Talon resin overnight. The resin was loaded onto a gravity column, and non-specifically bound proteins were eluted with 30 column volumes (CVs) of wash buffer (20 mM HEPES, 250 mM NaCl, 0.1 μM TCEP, and 20 mM imidazole). The target protein was eluted using 16 CV of elution buffer (20 mM HEPES, 250 mM NaCl, 0.1 μM TCEP, and 300 mM imidazole). Final purification was achieved via size-exclusion chromatography (SEC) on a Superdex 200 10/300 GL column equilibrated with S200 buffer (20 mM HEPES, 200 mM NaCl, and 0.1 μM TCEP), yielding a major peak at ~14 mL. Purified GRK5 was concentrated to an optimal concentration for downstream applications.

The β-arrestin plasmids were transformed into BL21(DE3) cells and cultured in TB medium. Induction and harvesting followed the same protocol as for GRK5. Cells were lysed in buffer (50 mM HEPES, 500 mM NaCl, 15% glycerol, and 0.1 μM TCEP), sonicated, and centrifuged. The supernatant was incubated with Talon resin overnight. After loading onto a gravity column, impurities were removed sequentially with 20 CV of wash buffer 1 (20 mM HEPES, 500 mM NaCl, 10% glycerol, 0.1 μM TCEP, and 20 mM imidazole) and 20 CV of wash buffer 2 (40 mM imidazole). The target protein was eluted with 10 CV of elution buffer (20 mM HEPES, 500 mM NaCl, 5% glycerol, 0.1 μM TCEP, and 250 mM imidazole). SEC purification was performed using S200 buffer (20 mM HEPES, 150 mM NaCl, 0.1 μM TCEP), with the major peak eluting at ~14 mL. The purified β-arrestin protein was concentrated and stored.

Expression of GRK2/3/6 was carried out in *Sf*9 insect cells cultured in ESF921 medium (Expression Systems, Davis, CA, USA) until reaching a density of ~2.1 × 10^6^ cells/mL. Cells were infected with recombinant baculovirus at a multiplicity of infection (MOI) = 5. After 48 h, cells were harvested by centrifugation. Protein purification protocols, including lysis, affinity chromatography, and SEC, were identical to those described for GRK5.

### 4.4. AT1R Phosphorylation In Vitro

The phosphorylation reaction system was prepared by calculating the equal molar ratios of GRK and AT1R proteins. Two distinct reaction buffers were formulated: Phosphorylation Buffer 1 (20 mM HEPES, 35 mM NaCl, 5 mM MgCl_2_, 50 μM TCEP, 200 μM ATP, and 50 μM ligand) for GRK5/6-mediated phosphorylation and Phosphorylation Buffer 2 (20 mM Tris-HCl, 6 mM MgCl_2_, 1 mM EDTA, 200 μM ATP, and 50 μM ligand) for GRK2/3-mediated phosphorylation. Purified GRK and AT1R proteins were added to their respective buffers, thoroughly mixed, and incubated at 30 °C for 1 h. Following the phosphorylation reaction, samples were collected for Western blot analysis to quantify the total and phosphorylated AT1R proteins.

### 4.5. Western Blots

To assess the phosphorylation status of AT1R, the pIMAGO assay kit (Sigma-Aldrich, St. Louis, MO, USA) was used according to the manufacturer’s instructions. Following standard SDS-PAGE, proteins were transferred to polyvinylidene difluoride (PVDF) membranes at 4 °C for 2 h. Membranes were blocked with blocking buffer at room temperature (RT) for 1 h, and subsequently incubated overnight at 4 °C with pIMAGO antibody (1:4000). After washing, membranes were incubated with avidin–horseradish peroxidase (HRP) secondary antibody for 1 h at RT. For total Flag–AT1R detection, equal volumes of sample were loaded per lane. Membranes were blocked with 5% bovine serum albumin (BSA) in TBST (Tris-buffered saline containing 0.1% Tween 20) at RT for 1 h, followed by overnight incubation at 4 °C with mouse-derived monoclonal anti-Flag M2 peroxidase HRP antibody (1:8000, HUABIO, Hangzhou, China). Detection was performed using a goat-anti-mouse-IgG-HRP secondary antibody for 1 h at RT. Immunoreactive bands were visualized using an Azure 600 imager (Azure Biosystems, Dublin, CA, USA).

### 4.6. Pull-Down Assays

The phosphorylated AT1R was incubated with β-arrestin at a 1.5-fold molar ratio at 25 °C for 30 min, followed by a 3 h incubation with amylose resin to perform MBP pull-down assays. The resin was loaded into a gravity column, and after complete drainage of the flow-through, unbound proteins were removed using 10 column volumes (CVs) of wash buffer (20 mM HEPES, 100 mM NaCl, 0.002% (*w*/*v*) LMNG, 2 mM MgCl_2_, and 10 μM ligand). Subsequently, AT1R and its bound β-arrestin complex were eluted with 10 CV of elution buffer (20 mM HEPES, 100 mM NaCl, 0.002% (*w*/*v*) LMNG, 2 mM MgCl_2_, 50 μM ligand, and 2 mM maltose). The binding stoichiometry of β-arrestin was directly analyzed by SDS-PAGE.

### 4.7. Molecular Dynamic Simulations

We build an all-atom model of AT1R comprising residues 11–359. The Sar^l^-AngII-activated AT1R structure (PDB ID: 7F6G) was employed, with Chain A (AT1R) and Chain B (Sar^1^-AngII) extracted for modeling. CT residues 320–359 were modeled by the AlphaFold3 server with default settings [48]. Four systems were modeled: (1) non-phosphorylated state, (2) Motif I-phosphorylated, (3) Motif II-phosphorylated, and (4) dual phosphorylation at both Motif I/II. All phosphorylation modifications were introduced directly through CHARMM-GUI [53] by patching SER and THR residues at designated positions with SEP and TPO residues. The CHARMM-GUI builder was used to (a) construct a lipid bilayer with a POPC: cholesterol ratio of 9:1, (b) embed the AT1R into the bilayer (the receptor orientation was aligned based on the OPM database), (c) neutralize the global electrostatic charge, and (d) adjust the ionic strength with 0.15 M NaCl. To reflect human physiological conditions, each solvated system was first equilibrated in NVT for 500ps in 310K. In this step, the protein and lipid bilayer were restrained in their positions using a harmonic restraining force. Following system setup, the simulation system was equilibrated in the NPT ensemble for 10 ns, with positional restraints applied to the heavy atoms of the protein. These restraints were gradually released throughout the equilibration phase. The final equilibrated structure was used as the starting conformation for production simulations. Each system was simulated in triplicate with different initial velocity distributions, and each replica was run for 200 ns. All simulations were carried out using GROMACS v2021.5 [54]. The CHARMM36m force field [55] was chosen for its accurate performance on membrane proteins and disordered regions. Parameters for ligand NAG and modified residue SAR were generated with the CHARMM General Force Field [56]. All MD trajectories (~200 ns × 3 × 4 systems = 2.4 μs total) were computed using a high-performance workstation equipped with 2 NVIDIA GeForce RTX 4090 GPUs.

### 4.8. AlphaFold3 Structure Prediction

The AF3 server was utilized (https://alphafoldserver.com/) to predict the AT1R-CT (320–359)-β-arrestin complex models. The sequences submitted to the server corresponded to the UniProt entries (AT1R: P30556, β-arrestin 1: P32121, and β-arrestin 2: Q9Y5N1). Structure prediction of the phosphorylated Motif I/II-containing AT1R-CT in complex with β-arrestin1/2 variants harboring constitutive activating mutations was performed using AlphaFold3. All components of the complex were included in the prediction to model the full assembly. The random seed was set to its default value, and the top-ranked predicted structure, based on confidence metrics, was selected for subsequent comparative analysis.

## Data Availability

The original contributions presented in this study are included in the article/Supplementary Material. Further inquiries can be directed to the corresponding authors.

## Supplementary Materials

The following supporting information can be downloaded at: https://www.mdpi.com/article/10.3390/ijms26167988/s1. Refs. [57,58] exist in Supplementary Materials.
