# Supplementary material for: Distinct Phosphorylation Patterns of AT1R by Biased Ligands and GRK Subtypes
Source: Int J Mol Sci. 2025 Aug 19;26(16):7988. doi: 10.3390/ijms26167988 (PMC12386296; doi:10.3390/ijms26167988)
Supplement: Supplementary file 1 [file ijms-26-07988-s001.zip › ijms-3795954-supplementary.pdf]

Supplementary Materials

Supplementary Figures

Supplementary Figure 1

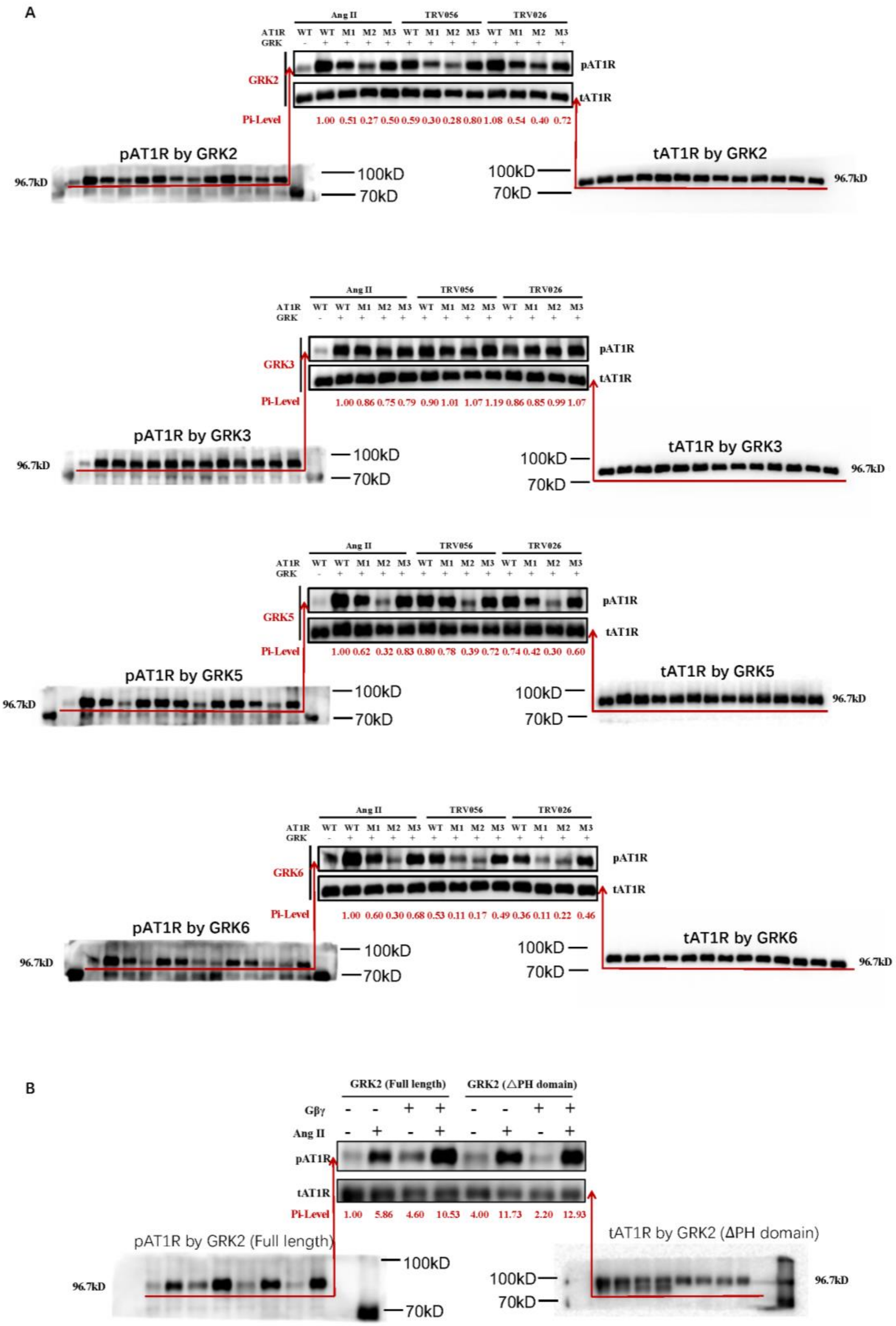

Vazyme, 180kDa Prestained Protein Marker, MP102-02-AA

Supplementary Figure 2

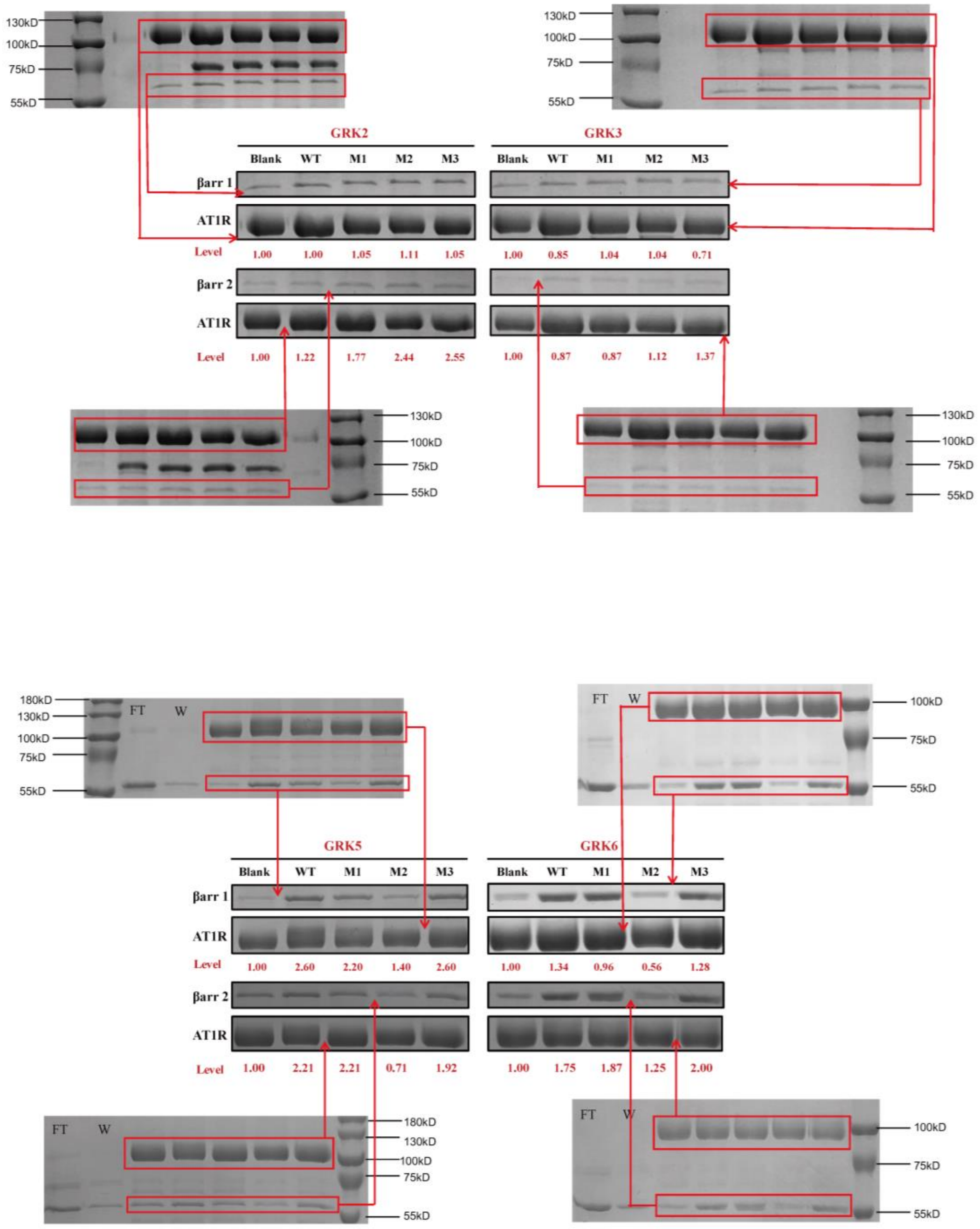

Vazyme, 180kDa Prestained Protein Marker, MP102-02-AA

Supplementary Methods

1. Model construction

We extracted Chain A (AT1R) and Chain B (Sarl-Ang II) from the cryo-EM structure PDB ID: 7F6G and exclude other components (Gq proteins). The missing C-terminal residues (320–359) in PDB were modeled using AlphaFold3 with default settings, and phosphorylation modifications were introduced using CHARMM-GUI by patching serine and threonine residues to SEP (phosphoserine) and TPO (phosphothreonine) at defined positions (Motif I and/or II). The receptor was then embedded into a POPC/cholesterol (9:1) bilayer using the CHARMM-GUI Membrane Builder. The receptor orientation was aligned based on the OPM database

2. Justification for parameter selection

We selected the CHARMM36m force field due to its proven performance in simulating GPCRs and intrinsically disordered regions such as the AT1R C-terminal tail. The simulation temperature (310 K) and ionic strength (0.15 M NaCl) were chosen to mimic physiological conditions. The CHARMM General Force Field (CGenFF) was used to parameterize non-standard ligands (e.g., NAG and SAR).

The simulation parameters and protocol we adopted are consistent with those broadly used and validated in recent studies of GPCR simulation [1][2], which supports the robustness and reproducibility of our MD simulations.

3. Experimental conditions

We now provide a clear description of the equilibration and production simulation protocol. Each solvated system was first equilibrated in NVT for 500 ps at 310 K. In this step, the protein and lipid bilayer were restrained in their positions using a harmonic restraining force. We used a velocity-rescaling thermostat and applied positional restraints of 1000 kJ/mol/nm^2 to all heavy atoms. Following system setup, the simulation system was equilibrated in the NPT ensemble for 10 ns, with positional restraints applied to the heavy atoms of the protein. These restraints were gradually released throughout the equilibration phase. Pressure was maintained at 1 bar using a semi-isotropic Parrinello–Rahman barostat. Bond lengths involving hydrogen atoms were constrained using the LINCS algorithm, and a 2 fs integration time step was used.

The final equilibrated structure was used as the starting conformation for production simulations. Each system was simulated in triplicate with different initial velocity distributions, and each replica was run for 200 ns.

4. Computational resource usage

Simulations were conducted on a high-performance workstation equipped with an AMD Ryzen 9 7950X CPU and 2 NVIDIA RTX 4090 GPUs. Each 200 ns trajectory required approximately 24–36 hours of wall time. The total simulation time across 12 trajectories (4 systems × 3 replicas) amounted to 2.4 μs.

[1]. Aranda-García, D., Stepniewski, T.M., Torrens-Fontanals, M. et al. Large scale investigation of GPCR molecular dynamics data uncovers allosteric sites and lateral gateways. Nat Commun 16, 2020 (2025).
[2]. Powers, A.S., Khan, A., Paggi, J.M. et al. A non-canonical mechanism of GPCR activation. Nat Commun 15, 9938 (2024).
